# Supplementary material for: Misadjustment of diurnal expression of core temperature and locomotor activity in lactating rabbits associated with maternal over-nutrition before and during pregnancy
Source: PLoS One. 2020 May 8;15(5):e0232400. doi: 10.1371/journal.pone.0232400 (PMC7209125; doi:10.1371/journal.pone.0232400)
Supplement: S1 Table — (PDF) [file pone.0232400.s001.pdf]

S1 Table.- Organs weights.

	light phase		dark phase		*p* values		
**Organs**	**SD**	**HFCD**	**SD**	**HFCD**	**MN**	**ZT**	**MN x ZT**
**brain**	1.5±0.1	1.7±0.1	1.4±0.09	1.4±0.1	0.83	0.13	0.34
**liver**	3.1±0.1	3.3±0.3	3.1±0.1	3.18±0.1	0.64	0.68	0.90
**kidney**	1±0.1	1±0.08	0.9±0.03	0.9±0.05	0.89	0.62	0.87
**heart**	0.5±0.02	0.5±0.02	0.4±0.03	0.5±0.04	0.54	0.07	0.39
**stomach**	5.7±0.3	4.9±0.4	7.1±1.4	6.8±0.7	0.52	0.05*	0.76
**BAT**	0.3±0.06	0.4±0.04	0.3±0.03	0.3±0.05	0.25	0.15	0.44
**mWAT**	0.3±0.07	0.4±0.02	0.4±0.04	0.5±0.08	0.31	0.08	0.87
**rWAT**	0.2±0.04	0.4±0.04	0.4±0.05	0.5±0.2	0.15	0.13	0.88

Normalized organs weight (g) of rabbits pups obtained from females fed standard diet (SD) or high fat and carbohydrate diet (HFCD), at the end of the lactancy. Results of one-way ANOVA obtained for differences associated with maternal nutrition (MN) and the time (zeitgeber time, ZT). Brown adipose tissue (BAT), mesenteric (mWAT) and retroperitoneal (rWAT) white adipose tissue. Mean ± SEM.
